# Long noncoding RNA PANDAR inhibits the development of lung cancer by regulating autophagy and apoptosis pathways

**DOI:** 10.7150/jca.45291

**Published:** 2020-06-06

**Authors:** Lan Zhang, Yuanhang Wang, Shengyuan Xia, Lei Yang, Di Wu, Yifeng Zhou, Jiachun Lu

**Affiliations:** 1 Department of Medical Genetics and Cell Biology, GMU-GIBH Joint School of Life Sciences, Guangzhou Medical University, Guangzhou, China.; 2 Jiaxing Center for Disease Control and Prevention, Jiaxing, China.; 3 The State Key Lab of Respiratory Disease, The First Affiliated Hospital, The institute for Chemical Carcinogenesis, School of Public health, Guangzhou Medical University, Guangzhou, China.; 4 Guangzhou Center for Disease Control and Prevention, Guangzhou, China.; 5 Department of Genetics, Medical College of Soochow University, Suzhou, China.

**Keywords:** PANDAR, BECN1, lung cancer, autophagy, apoptosis

## Abstract

**Background:** LncRNAs has been shown to play important roles in the progression of lung cancer, but it remains poorly understood whether lncRNAs affect the occurrence and development of lung cancer by regulating autophagy and apoptosis levels. Here, we investigated the roles of PANDAR in NSCLC.

**Materials and Methods:** The expression profile and clinical application of PANDAR and its possible target gene BECN1 were tested in 276 cases of lung cancer tissues. Through some actual experiments, we explored functions of PANDAR about proliferation, apoptosis and autophagy of NSCLC cells *in vitro*.

**Results:** PANDAR was found to downregulate both in lung cancer tissues and cell lines compared with corresponding controls (P < 0.05 for all), which was related to tumor stage (P < 0.05). Moreover, autophagy related gene BECN1 was also downregulated in lung cancer tissues comparison with normal tissues (*P* < 0.01), and there was a significant positive correlation between PANDAR and BECN1 levels (r = 0.789, *P* < 0.001). So, the high expression of PANDAR increased BECN1 expression levels and impaired the proliferation of NSCLC cell lines *in vitro*. Furthermore study showed PANDAR could regulate cell autophagy and apoptosis levels.

**Conclusion:** These results indicated lncRNA PANDAR was a tumor suppressor and can inhibit NSCLC cell proliferation by activating autophagy and apoptosis pathways via upregulation of BECN1 expression.

## Introduction

Lung cancer remains the main factor of deaths due to carcinoma over the years^1^. The leading reason for poor treatment of lung cancer is many cases have reached an advanced stage 2. NSCLC was nearly 80 per cent among lung cancer patients and makes up to the majority of cancer deaths 3. Although, survival rate of lung cancer has improved with the development in chemotherapy, surgical techniques and molecular targeted therapies, the 5-year survival rate of advanced patients is still less than 5% 4. The reason for that is the unclear molecular mechanism. Therefore, a detailed elaboration on the mechanisms is indispensable for improving the diagnosis, prevention and treatment of NSCLC.

Autophagy is an intracellular catabolic and recycled process with lysosome-mediated 5. Theoretically, autophagy promotes survival by eliminating cells of toxic metabolites, damaged organelles, intracellular pathogens or by excessive self-digestion and degradation of elementary cell compositions, but it is not known whether autophagy regulates cellular survival or death pathway or both 6. So far, researchers have indicated that autophagy is closely associated with the progression of NSCLC 7, 8. For example, BECN1 with encoding Beclin1 protein is a specific gene for autophagy 9. Numerous studies have indicated that BECN1 is not only involved in the formation of autophagosome, but also influences the development of lung cancer by regulating cell autophagy levels 10.

LncRNA has no protein-coding ability but it could regulate gene expression 11. It involves in biological processes of diverse cancers including NSCLC 12-14. For example, some papers have revealed that lncRNAs are closely related to the formation, development, migration and metastasis of NSCLC, particularly MEG3, HOTAIR, FENDRR and others 15-17. Moreover, some studies have concluded that lncRNAs expression patterns in NSCLC are involved in the expression of thousands of genes concurrently 18, 19. LncRNAs can act a pivotal part in NSCLC progression through a great deal of signaling pathways 20. However, to date, the mechanisms and molecular pathways in NSCLC are not largely elusived. Therefore, identification of NSCLC-associated lncRNAs and investigation of the functions and mechanism are useful for improving diagnosis and treatment in NSCLC. Lately, Hung et al. have proved that PANDAR is interacted with transcription factor to limit pro-apoptotic genes expression 21. Han et al. concluded the low expression PANDAR can predict poor prognosis and affected apoptosis by regulating Bcl-2 in NSCLC 22. Pattingre et al. clarified the Bcl-2 functioned as an anti-apoptotic and anti-autophagy protein via interaction with Beclin1 23. Our early research results have demonstrated that the Beclin1 protein expression decreased in malignant transformation of 16HBE cells, indicating that autophagy acted a critical part in the development of lung cancer 24. Nowadays majority of evidences emphasize the roles of lncRNAs by the apoptotic pathways in cancer. There is little report about the relationship between lncRNAs and cell autophagic function in NSCLC. Thus, it is explored whether lncRNA PANDAR can play a significant biological role by regulating BECN1 expression and investigated potential mechanisms of PANDAR in NSCLC.

In this research, we investigated the expression profile and clinical application of lncRNA PANDAR and possible target gene BECN1 in lung cancer tissues. Experiments were performed to test the functions of lncRNA PANDAR on proliferation, apoptosis and autophagy of NSCLC cells* in vitro*.

## Materials and Methods

### Tissue collection

All subjects were Han Chinese in China. 276 lung cancer tissues and normal lung tissues were gathered from patients with pathologically diagnosed primary lung cancer. The cases had not received chemotherapy, radiation or immunotherapy prior to surgery. Among these, 182 samples were obtained from the First Affiliated Hospital and Cancer Center of Guangzhou Medical University, the Cancer Hospital of Kunming Medical University in southern China. 94 samples were collected from the First Affiliated Hospital of Soochow University in eastern China. All samples involved in the study had no genetic relationship with each other. All tissues were stored at -80℃ in liquid nitrogen.

### Cell lines and culture conditions

The 16HBE and human NSCLC cell lines (L78, PC9, 95D, NCI-H460, A549) were purchased from Shanghai Institute of Cell Biology. These cells were cultured in RPMI-1640 medium with 10 per cent FBS and penicillin/streptomycin with 5 per cent carbon dioxide at 37℃.

### RNA extraction and qRT-PCR

Total RNA was extracted from tissues and cells using TRIzol reagent. First-strand cDNA was synthesized with PrimeScript RT reagent Kit. qRT-PCR were used to detecte the expression of PANDAR and BECN1 using SYBR Green Mixture with ROX, BIOMIGA and gene specific primers. Data was collected on ABI 7900. β-actin was selected as an internal reference gene to normalize results, and all primer sequences were as follows. β-actin (forward: GGCGGCACCACCATGTACCCT; reverse: AGGGGCCGGACTCGTCATACT), PANDAR (forward: TCCCAACAAACAAGGGGTGG; reverse: GTGGCCAAAGGATCTGACGA), BECN1 (forward: TGGCACAATCAATAACTTCAGG; reverse: GACCCATCTTATTGGCCAGA), GAPDH (forward: AGCCACATCGCTCAGACAC; reverse: GCCCAATACGACCAAATCC), U6 (forward: CTCGCTTCGGCAGCACA; reverse: AACGCTTCACGAATTTGCGT).

### Subcellular fractionation

To explore cellular localization of PANDAR, cytosolic and nuclear fractions were collected using nuclear/cytoplasmic isolation kit. We examined the expression of PANDAR using qRT-PCR, GAPDH and U6 in cytoplasm and nuclear fractions. GAPDH and U6 were used as cytosolic and nuclear fraction indicators respectively.

### Plasmid construction

The sequence of PANDAR control were synthesized and subcloned into pEZ-Lv206 from GeneCopoeia, Guangzhou, China. The ectopic expression of PANDAR was obtained using the pEZ-Lv206-PANDAR transfection, and empty pEZ-Lv206 particles was used as a control group. The resulting construct was verified by sequencing from Sangon Biotech, Shanghai, China.

### Cell transient transfection

Cell lines were transfected with pEZ-LV206-PANDAR, pEZ-LV206-control and extracted with Plasmid Midiprep System, Promega. PC9 and A549 cells were cultured for 24 h and transfected into six-well plate using Lipofectamine3000. The transfected cell lines were harvested after 48 h or 72 h for follow-up series of cell assays. Besides, the experimental methods of two plasmids co-transfection were same as above.

### Western blot analysis and antibodies

Western blot analysis was used for examining protein expression. The cells were collected and lysed using RIPA buffer supplemented with protease inhibitors. BECN1 and LC3 were the detecting antibodies. GAPDH was used as the housekeeping gene. All antibodies, including Beclin1, LC3, GAPDH and HRP-linked secondary antibody (Rabbit) were from CST.

### Cell proliferation assays

The effects of upregulated PANDAR about proliferation in A549 and PC9 cells were tested with CCK8. 24 hours after transfection with pEZ-LV206-PANDAR or control, cells were collected and seeded 1000 cells per well in 96-well plates. From a period, CCK8 was added in at least five replicate wells and incubated for 1-4 hours at 37℃. OD was measured at 450 nm by a plate reader.

### Morphological examination of apoptotic cells

PC9 and A549 were treated as indicated and incubated for 48 hours, and stained with Hoechst 33258 for 30 min at 37°C in the dark. Fluorescence images of normal and apoptotic cells were examined under fluorescence microscope.

### Detection of BECN1 release

In order to determine the main pathway of PANDAR inducing apoptosis by regulating BECN1 expression, PC9 and A549 cells were co-transfected with DsRed-Mit and GFP-BECN1. The experimental group cells were treated by apoptosis promoter EBSS (Sigma-Aldrich, Germany), and the control group cells did not any treatment, and then imaged by the fluorescence microscope. We obtained images of GFP-BECN1 and DsRed-Mit respectively, and then merged them. The images of GFP-BECN1 and DsRed-Mit were obtained separately and then merged. The BECN1 released from mitochondria was determined based on the overlap of GFP-BECN1 and DsRed-Mit fluorescence images.

### Statistical analysis

T-test was used to evaluate the discrepancy in gene expression between two groups Statistical analysis was conducted with Stata 12.0 software. A two sided value of *P*<0.05 was considered having statistical significance.

## Results

### PANDAR expression is down-regulated in human lung cancer tissues

**Figure 1:**
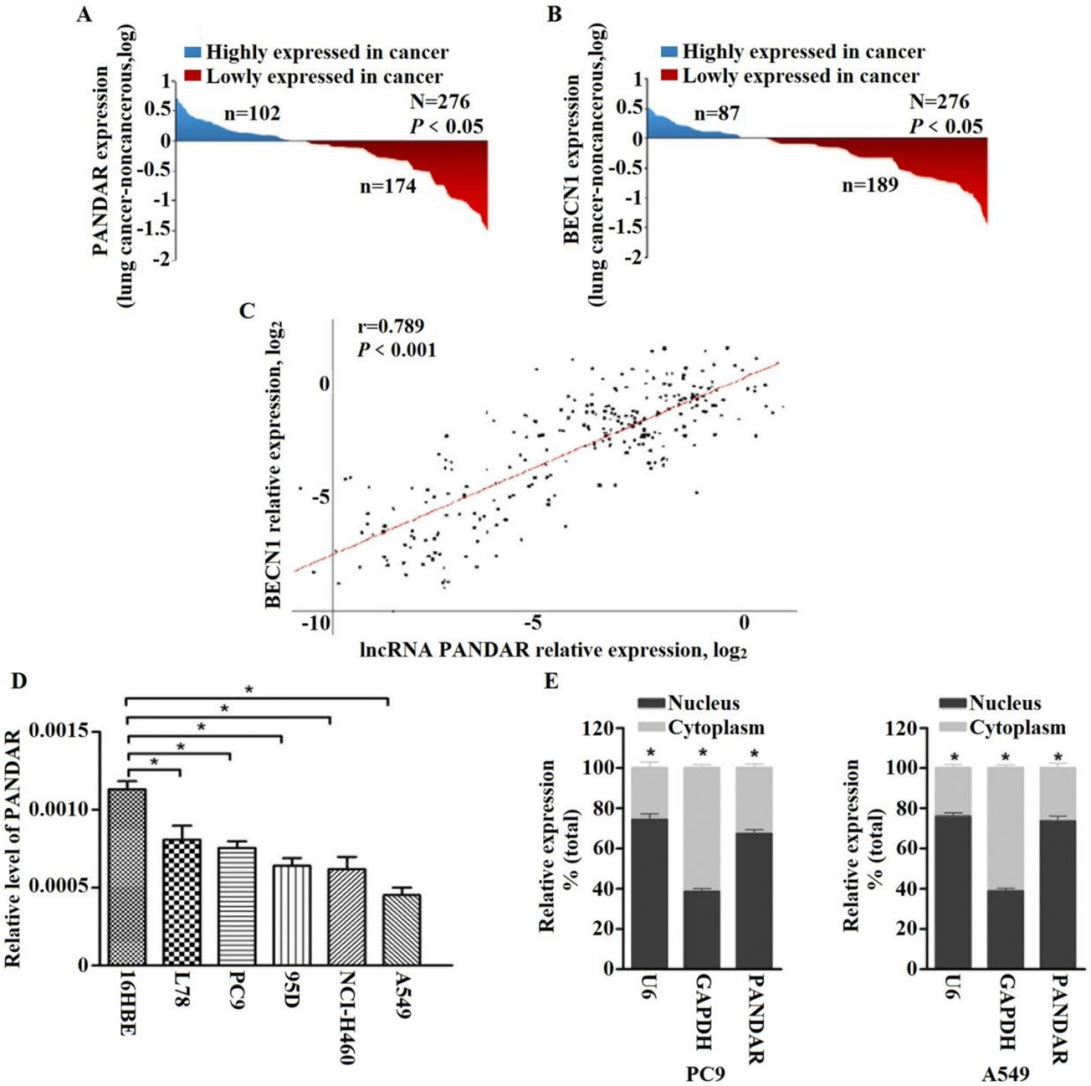
** PANDAR expression is down-regulated in human lung cancer tissues and cell lines. (A)** The analysis of the PANDAR expression levels was performed in 276 pairs of lung cancer tissues by qRT-PCR and normalized to βactin expression. The expression of PANDAR in lung cancer tissues is lower than those in non-tumorous tissues. **(B)** Comparing differences in the expression levels of BECN1 between tumor and corresponding normal tissues (n=276). **(C)** Positive correlation between PANDAR and BECN1 mRNA expression levels in 276 pairs of lung cancer tissue samples (r=0.789, *P*<0.001).** (D)** Levels of PANDAR in NSCLC cell lines and the normal pneumonocyte cell line 16HBE. Mean ± SD represents three independent experiments (*P < 0.05). **(E)** PANDAR nuclear localization, as identified using qRT-PCR in fractionated PC9 and A549 cell lines. GAPDH was used as a cytosol marker and U6 was used a nucleus marker. Mean ± SD represents three independent experiments (*P < 0.05).

To clarify the roles of PANDAR in NSCLC development, we explored the PANDAR expression status in tissues and found the expression of PANDAR in cancer tissues was downregulated compared to normal tissues (*P* < 0.05) (Fig. 1A). Meanwhile, the expression of autophagy related gene BECN1 mRNA expression was assayed and found that the BECN1 mRNA expression in cancer tissues was remarkably lower than that in normal tissues (*P* < 0.01) (Fig. 1B). Importantly, a positive association was observed between PANDAR and BECN1 in lung cancer tissues (r = 0.789, *P* < 0.001) (Fig. 1C). It demonstrated PANDAR may be associated with BECN1 gene in NSCLC.

### Associations between PANDAR expression and lung cancer progression

According to the expression status of lncRNA PANDAR in tissues, it can be divided into “High” or “Low” expression group. As listed in Table 1, the levels of PANDAR expression was notable difference at TNM stages (*P* = 0.001), and lower status of PANDAR expression were observed at advanced T status (T3 + T4) than those with early T status (T1 + T2) (*P* = 0.001). Low expression of PANDAR was related to risk of lung cancer progression when merged the two characteristics. However, no other association was observed.

### PANDAR expression status in cell lines

To determine the function of PANDAR in NSCLC, we analyzed the expression levels of PANDAR in human NSCLC and 16HBE cells. Fig. 1D revealed that the expression of PANDAR were markedly downregulated in NSCLC cells (L78, PC9, 95D, NCI-H460 and A549) compared with 16HBE (*P* < 0.05). Based on the qRT-PCR results, we selected two representative cells (PC9 and A549) for subsequent cell functional experiments *in vitro*.

### Subcellular fractionation location

To investigate the cellular localization of PANDAR, we fractionated PC9 and A549 into nuclear and cytoplasmic fractions. The results revealed that PANDAR was strongly enriched in the nuclear fraction of both PC9 and A549 cells (*P* < 0.05) (Fig. 1E), thus indicating PANDAR plays a regulatory role at the transcriptional levels.

### Upregulated PANDAR increases the expression of BECN1

**Figure 2:**
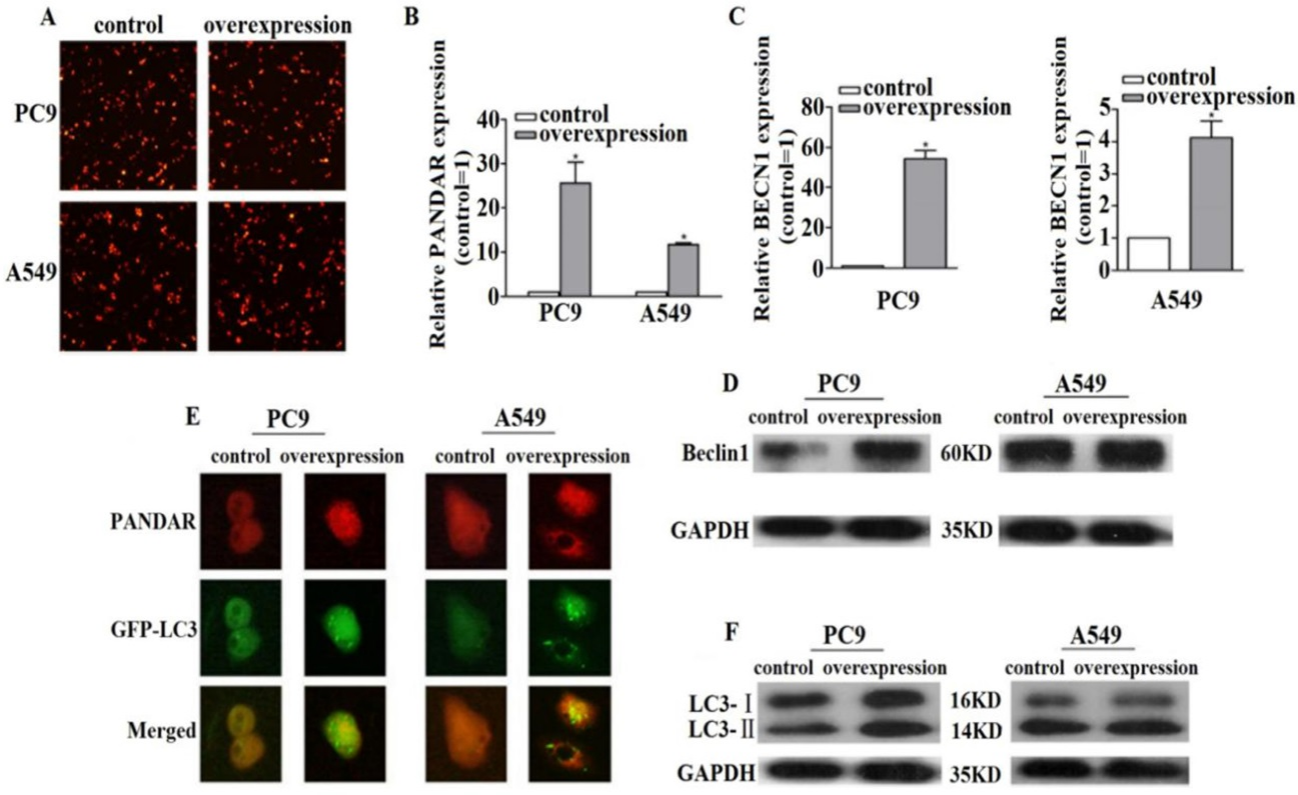
** The relationship between lncRNA PANDAR and BECN1 mRNA. (A)** After transfecting pEZ-LV206-PANDAR or pEZ-LV206-control, we could preliminary judge the transfection efficiency of plasmid by fluorescence microscope. **(B)** PC9 and A549 cells were treated with pEZ-LV206-PANDAR or pEZ-LV206-control, and PANDAR expression was assayed by qRT-PCR. **(C)** qRT-PCR results showed that PANDAR overexpression resulted in an increase of the BECN1 mRNA expression levels in PC9 and A549 cells. **(D)** Western blot analysis of Beclin1 protein was performed at 72h. The results are presented as the Mean ± SD (*P<0.05). **(E)** The endogenous punctate structures represented relative autophagy levels by detecting fluorescence images of PANDAR and GFP-LC3 after transfection.** (F)** Western blot analysis was applied to elucidate the expression levels of LC3-Ⅱ protein (autophagy marker). Data are representative of three independent experiments.

To determine the function of PANDAR, we evaluated the effects of PANDAR overexpression. The qRT-PCR results manifested PANDAR expression was increased both in PC9 and A549 cells after transfection with pEZ-Lv206-PANDAR (*P*<0.05) (Fig. 2A, 2B). Moreover, qRT-PCR results revealed the expression levels of BECN1 mRNA were increased when PANDAR was overexpressed compared with the pEZ-Lv206-control in PC9 and A549 cells (*P* < 0.05) (Fig. 2C). To further show the impact of PANDAR on BECN1 mRNA expression, western blot analysis was used to detect the expression levels of Beclin1 protein and the results exhibited the same trend (Fig. 2D).

### Overexpression of PANDAR induces autophagy levels in NSCLC

To explore the mechanisms of PANDAR in regulating NSCLC progression, we investigated whether PANDAR regulated BECN1 mRNA signals, focusing on autophagy. When cell autophagy was stimulated, GFP-LC3 redistributed from a diffuse cytoplasmic pattern to form punctate structures that label preautophagosomal and autophagosomal membranes. Results showed that representative fluorescent microscopy images of GFP-LC3 endogenous punctate structures treated with pEZ-Lv206-PANDAR were more than control groups in PC9 and A549 cells. The fluorescence microscopy analysis revealed that PANDAR overexpression increased autophagy levels (Fig. 2E). Western blot analysis further illustrated that PANDAR overexpression led to a markedly increase of LC3-II protein compared with control (Fig. 2F). These data suggested that upregulated PANDAR contributes to autophagy activation in NSCLC.

### Upregulated PANDAR inhibits cell proliferation by activating autophagy

**Figure 3:**
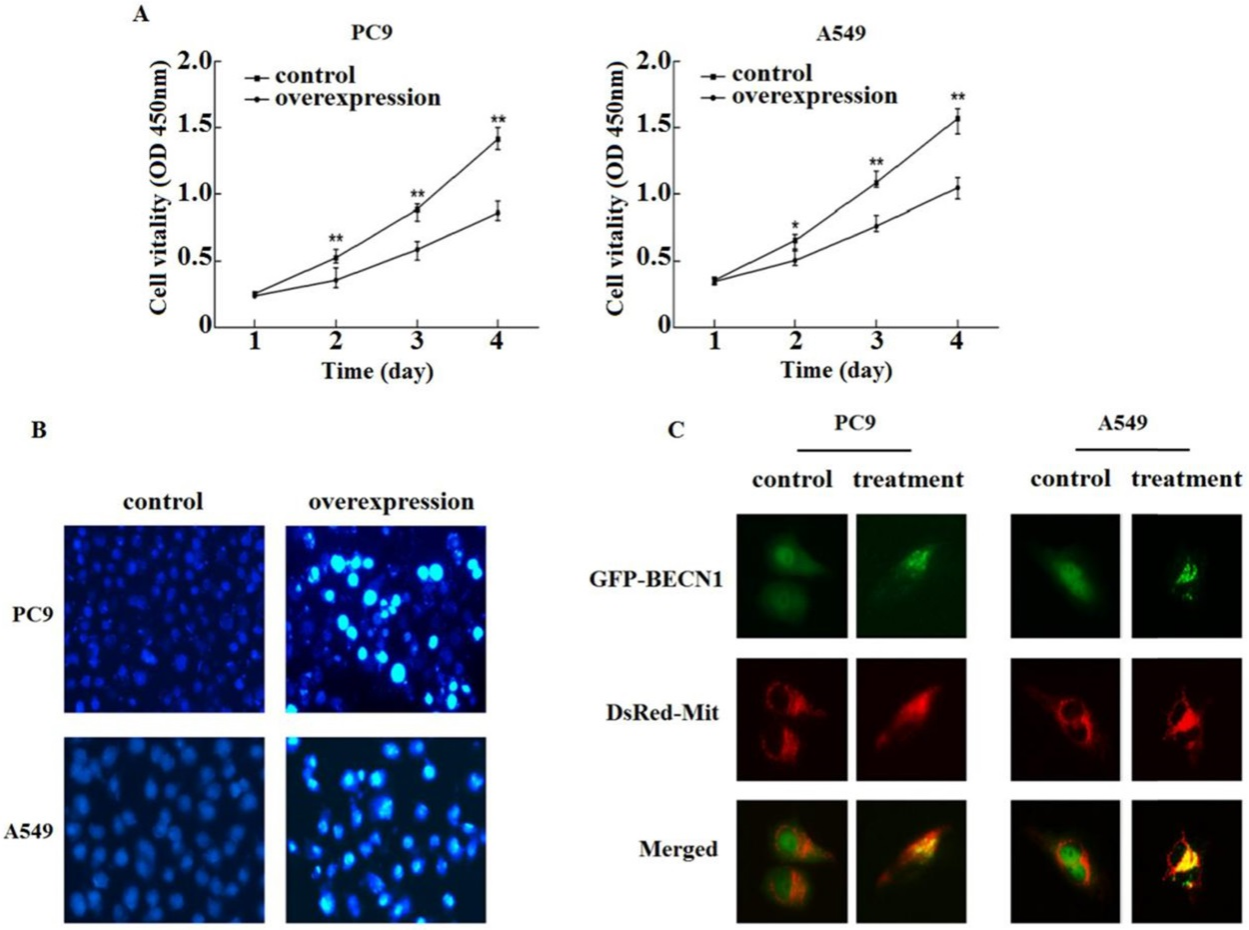
** PANDAR overexpression inhibited proliferation and promoted apoptosis. (A)** The roles of PANDAR in the regulation of cell proliferation. PC9 and A549 cells were treated with pEZ-Lv206-PANDAR or pEZ-Lv206-control. Cell proliferation was assayed by using CCK-8 according to the manufacture's protocol. Means±SD were shown (n=5). *P<0.05, **P<0.01 (vs.control). **(B)** Hoechst 33258 morphological examination of PANDAR promoting apoptosis. Typical apoptotic form after treating with pEZ-Lv206-PANDAR. Data are representative of three independent experiments. **(C)** Translocation of GFP-BECN1 treated with apoptosis promoter EBSS. PC9 and A549 cells transiently co-expressing GFP-BECN1 and DsRed-Mit. Translocation of GFP-BECN1 was performed by fluorescence microscopy. Control cells without GFP-BECN1 translocation over time. PC9 and A549 cells treated with EBSS, GFP-BECN1 translocated to mitochondria noticeably when cell apoptosis occured. Data are representative of three independent experiments.

To discuss the roles of PANDAR in regulating proliferation by autophagy, PC9 and A549 were treated with pEZ-Lv206-PANDAR or control. The CCK8 assay illustrated the upregulated PANDAR inhibited the proliferation in comparison to control among PC9 and A549 (Fig. 3A). Therefore, we concluded that the upregulated PANDAR may inhibit NSCLC cells proliferation by activating autophagy levels.

### Hoechst 33258

To evaluate cell apoptotic status, morphological examinations were performed. We observed chromatin condensation in the PC9 and A549 with pEZ-Lv206-PANDAR or control respectively with Hoechst 33258. The nucleus of PC9 and A549 treated with pEZ-Lv206-PANDAR showed the occurrence of apoptosis in comparision with that of control (Fig. 3B). The results revealed upregulated PANDAR could promote NSCLC apoptosis.

### Detection of Beclin1 release during PANDAR-induced apoptosis

In order to understand this apoptotic pathway, we further observed the release of Beclin1 protein from mitochondria to the cytoplasm in PC9 and A549 co-expressed with DsRed-Mit and GFP-Beclin1. Using fluorescence microscope and subcellular fractionation methods, the pattern of GFP-Beclin fluorescence treated with EBSS was showed it was no difference from that of DsRed-Mit (Fig. 3C). The confocal images authenticated the distribution of GFP-Beclin1 fusion protein was in the mitochondria when cell apoptosis occurred.

## Discussion

Recent studies have found lncRNA PANDAR interacted with transcription factor in normal human embryonic lung fibroblasts to inhibit the expression of pro-apoptotic genes. Subsequently, Han et al. confirmed that lncRNA PANDAR is closely related to the prognosis in lung cancer, which can affect cell apoptosis by regulating Bcl-2. Bcl-2 was an anti-apoptotic protein, and could regulate cell autophagy levels by which interacted with Beclin1 to form Beclin1-Bcl-2 complex through BH3 domain. Furthermore, we have confirmed that the Beclin1 expression was decreased in malignant transformed 16HBE cells, indicating that autophagy plays significant role in lung cancer. Therefore, we deduced lncRNA PANDAR should be associated with BECN1, and PANDAR involved in the development of lung cancer by regulating autophagy pathway in lung cancer. The PANDAR more likely functions as a novel tumor suppressor.

In this study, we concluded the expression of lncRNA PANDAR in 276 lung cancer tissues were lower than that in the matched normal lung tissues. The high expression of PANDAR was significantly associated with TNM stage and primary tumor depth of invasion. While BECN1 was in a lower expression in lung cancer tissues than in normal lung tissues, there was a positive correlation relationship between PANDAR and BECN1. In order to further illustrate the relationship between lncRNA PANDAR and BECN1, we established pEZ-Lv206-mediated overexpression of PANDAR in PC9 and A549 cells. We found overexpressed PANDAR would result in the expression levels of BECN1 gene and its corresponding protein Beclin1 were both increased obviously by qRT-PCR and Western Blot experiments. These results suggested that lncRNA PANDAR can promote BECN1 expression at the transcription and translation levels in lung cancer. At present, studies have revealed BECN1 was one of the most important genes involved in autophagy. Therefore, we investigated the relationship between lncRNA PANDAR and autophagy in lung cancer by PANDAR and GFP-LC3 plasmids co-transfection experiments, and the results demonstrated the GFP-LC3 endogenous punctate structures increased significantly in overexpressed PANDAR groups compared with control groups. In addition, the autophagy marker LC3-Ⅱ protein expression levels were also increased when PANDAR expression up-regulated, and this further validated the results of co-transfection experiments indicating that lncRNA PANDAR could increase autophagy levels in lung cancer cells. The possible mechanism was PANDAR involved in autophagy activation by up-regulating the expression levels of BECN1. To elucidate the biological roles of lncRNA PANDAR in the regulation of autophagy in NSCLC, cell viability assays were used and results showed optical density at PANDAR overexpression group was lower than control. These suggested that increased expression of lncRNA PANDAR could significantly inhibit the growth and proliferation of lung cancer cells and lncRNA PANDAR maybe inhibit the progression of lung cancer through autophagy pathway.

Many studies have confirmed that the process of autophagy and apoptosis occurred in tumor cells at the same time. Hoechst 33258 experiments were used to elucidate the relationship between lncRNA PANDAR and apoptosis in lung cancer cells, and the results showed in comparision with control group, the number of apoptotic lung cancer cells increased in overexpressed PANDAR group. So, we concluded that lncRNA PANDAR regulated the development of lung cancer involved in not only autophagy pathway but also apoptosis pathway. Mitochondrial apoptosis is one of the most important apoptotic pathways. Some researchers have found that various apoptotic stimulus signals can induce Bcl-2 family apoptosis related proteins with BH3 domain and regulate cell apoptosis through mitochondrial pathway. Beclin1 and Bcl-2 family proteins both have BH3 receptor domain, so we speculated that lncRNA PANDAR would promote the apoptosis of lung cancer cells in mitochondria pathway by increasing Beclin1 expression levels. In order to verify the above hypothesis, we used fluorescence microscope and subcellular fractionation methods to detect the location of Beclin1 release during PANDAR-induced apoptosis in NSCLC. The results showed that Beclin1 protein appeared punctate aggregation and its location was indistinguishable from DsRed-Mit when the PC9 and A549 cells occurred apoptosis. These results indicated PANDAR promoted cell apoptosis through mitochondrial pathway in the development of NSCLC. The possible mechanism was the up-regulated Beclin1 protein went into mitochondria, and affected the expression levels of apoptosis-related protein, such as Bax, thereby triggering apoptotic signals and promoting cell apoptosis.

In conclusion, this study has confirmed that lncRNA PANDAR can affect the development of lung cancer by regulating the expression of BECN1 gene. Further studies found PANDAR involved in autophagic regulation at the levels of transcription and translation, which led to the loss of the survival advantage of tumor cells, thus inhibited cell growth and proliferation. While, we didn't perform related experiments to test the invasion and migration ability of PANDAR *in vitro* and we will continue to examine other biological functions about lncRNA PANDAR in the future. In addition, PANDAR can promote cell apoptosis through the mitochondrial pathway. These results concluded lncRNA PANDAR can play essential roles in the development of lung cancer through autophagy and apoptosis pathways.

## Figures and Tables

**Table 1:** The relationship between PANDAR expression and clinicopathological features of NSCLC

Characteristics	Southern Samples, N (%)		*P^b^*	Eastern Samples, N (%)			*P^b^*	Total, N (%)			*P^b^*
	Low	High		Low	High			Low		High	
**Age**											
<60	72(68.6)	33(31.4)	0.486	32(62.7)		19(37.3)	0.176	104(66.7)	52(33.3)		0.155
≥60	49(63.6)	28(36.4)		21(48.8)		22(51.2)		70(58.3)	50(41.7)		
**Gender**											
Female	37(71.2)	15(28.8)	0.259	16(57.1)		12(42.9)	0.861	53(66.3)	27(33.7)		0.434
Male	81(62.3)	49(37.7)		39(59.1)		27(40.9)		120(61.2)	76(38.8)		
**Family History**											
No	94(58.4)	67(41.6)	0.758	49(58.3)		35(41.7)	0.477	143(58.4)	102(41.6)		0.512
Yes	13(61.9)	8(38.1)		7 (70.0)		3 (30.0)		20(64.5)	11(35.5)		
**Smoking**											
No	31(46.3)	36(53.7)	0.181	19(63.3)		11(36.7)	0.244	50(51.5)	47(48.5)		0.062
Yes	65(56.5)	50(43.5)		48(75.0)		16(25.0)		113(63.1)	66(36.9)		
**Drinking**											
No	69(50.4)	68(49.6)	0.261	38(52.1)		35(47.9)	0.425	107(51.0)	103(49.0)		0.170
Yes	27(60.0)	18(40.0)		13(61.9)		8 (38.1)		40(60.6)	26(39.4)		
**TNM stage**											
Ⅰ+Ⅱ	38(53.5)	33(46.5)	**0.031**	17(58.6)		12(41.4)	**0.019**	55(55.0)	45(45.0)		**0.001**
Ⅲ+Ⅳ	77(69.4)	34(30.6)		53(81.5)		12(18.5)		130(73.9)	46(26.1)		
**T status**											
T1+T2	53(51.0)	51(49.0)	**0.034**	28(62.2)		17(37.8)	**0.009**	81(54.4)	68(45.6)		**0.001**
T3+T4	52(66.7)	26(33.3)		42(85.7)		7(14.3)		94(74.0)	33(26.0)		
**N status**											
N0	41(62.1)	25(37.9)	0.822	23(53.5)		20(46.5)	0.193	64(58.7)	45(41.3)		0.318
N1+N2+N3	74(63.8)	42(36.2)		34(66.7)		17(33.3)		108(64.7)	59(35.3)		
**M status**											
M0	81(62.8)	48(37.2)	0.588	39(65.0)		21(35.0)	0.250	120(63.5)	69(36.5)		0.256
M1	31(58.5)	22(41.5)		18(52.9)		16(47.1)		49(56.3)	38(43.7)		
**Histological Classification**											
Adenocarcinoma	47(54.7)	39(45.3)	0.737	26(63.4)		15(36.6)	0.831	73(57.5)	54(42.5)		0.900
Squamous Carcinoma	28(54.9)	23(45.1)		17(54.8)		14(45.2)		45(54.9)	37(45.1)		
Large Cell Lung Cancer	2 (33.3)	4 (66.7)		4 (80.0)		1 (20.0)		6(54.5)	5(45.5)		
Small Cell Lung Cancer	10(52.6)	9 (47.4)		5 (62.5)		3 (37.5)		15(55.6)	12(44.4)		
Others^a^	13(65.0)	7 (35.0)		6 (66.7)		3 (33.3)		19(65.5)	10(34.5)		
											

^a^ Large cell carcinoma, small cell carcinoma, and hybrid or undifferentiated carcinoma; **^b^** Pearson χ^2^.

## Abbreviations

NSCLC: Non-small cell lung cancer
PANDAR: promoter of CDKN1A antisense DNA damage-activated RNA
qRT-PCR: quantitative real-time PCR
LncRNA: long non-coding RNA
16HBE: normal bronchial epithelial cell line
FBS: fetal bovine serum
RIPA: radioimmunoprecipitation assay
CCK8: Cell Counting Kit-8
OD: Optical density
TNM: tumor-node-metastases
EBSS: Earle's balanced salt solution
